# Promoting Engagement With Smartphone Apps for Suicidal Ideation in Young People: Development of an Adjunctive Strategy Using a Lived Experience Participatory Design Approach

**DOI:** 10.2196/45234

**Published:** 2023-06-06

**Authors:** Daniel Z Q Gan, Lauren McGillivray, Mark Erik Larsen, Taylor Bloomfield, Michelle Torok

**Affiliations:** 1 Black Dog Institute University of New South Wales Sydney Australia

**Keywords:** eHealth, digital mental health, smartphone app, engagement, youth suicide prevention, qualitative methods, suicide, development, youth, mental health, support, user-centered, design, survey, interview, prototype, prevention, participatory design, mobile phone

## Abstract

**Background:**

Suicide among young people is a worrying public health concern. Despite this, there is a lack of suitable interventions aligned with the needs of this priority population. Emerging evidence supports the effectiveness of digital interventions in alleviating the severity of suicidal thoughts. However, their efficacy may be undermined by poor engagement. Technology-supported strategies (eg, electronic prompts and reminders) have been deployed alongside digital interventions to increase engagement with the latter. However, evidence of their efficacy is inconclusive. User-centered design approaches may be key to developing feasible and effective engagement strategies. Currently, no study has been published on how such an approach might be expressly applied toward developing strategies for promoting engagement with digital interventions.

**Objective:**

This study aimed to detail the processes and activities involved in developing an adjunctive strategy for promoting engagement with LifeBuoy—a smartphone app that helps young people manage suicidal thoughts.

**Methods:**

Development of the engagement strategy took place in 2 phases. The discovery phase aimed to create an initial prototype by synthesizing earlier findings—from 2 systematic reviews and a cross-sectional survey of the broader mental health app user population—with qualitative insights from LifeBuoy users. A total of 16 web-based interviews were conducted with young people who participated in the LifeBuoy trial. Following the discovery phase, 3 interviewees were invited by the research team to take part in the workshops in the design phase, which sought to create a final prototype by making iterative improvements to the initial prototype. These improvements were conducted over 2 workshops. Thematic analysis was used to analyze the qualitative data obtained from the interviews and workshops.

**Results:**

Main themes from the interviews centered around the characteristics of the strategy, timing of notifications, and suitability of social media platforms. Subsequently, themes that emerged from the design workshops emphasized having a wider variety of content, greater visual consistency with LifeBuoy, and a component with more detailed information to cater to users with greater informational needs. Thus, refinements to the prototype were focused on (1) improving the succinctness, variety, and practical value of Instagram content, (2) creating a blog containing articles contributed by mental health professionals and young people with lived experience of suicide, and (3) standardizing the use of marine-themed color palettes across the Instagram and blog components.

**Conclusions:**

This is the first study to describe the development of a technology-supported adjunctive strategy for promoting engagement with a digital intervention. It was developed by integrating perspectives from end users with lived experience of suicide with evidence from the existing literature. The development process documented in this study may be useful for guiding similar projects aimed at supporting the use of digital interventions for suicide prevention or mental health.

## Introduction

Suicide among young people is a significant global public health concern [1]. In Australia, suicide is the leading cause of death among individuals aged 15 to 24 years [2]. Though it is well recognized that young people have different and unique mental health needs compared to adults [3], there continue to be few resources and interventions that have been specifically designed for young people and that have evidence for preventing suicide-related outcomes [4]. Self-guided digital mental health interventions (DMHIs) delivered through smartphone apps or web-based platforms without human guidance have rapidly emerged as a potential low-cost and scalable model of care for young people experiencing suicidal ideation, self-harm, and mental health issues [5].

Findings from a recent meta-analysis of self-guided DMHIs for suicide prevention show that self-guided digital interventions directly targeting suicidal ideation are effective in reducing the severity of suicidal thoughts, although evidence specifically in relation to young people is still rare [6]. However, there is ample evidence indicating that low levels of user engagement with DMHIs have hindered the real-world uptake of these interventions [7,8], including DMHIs targeted at younger users [9,10]. In addition, poor engagement—stopping use of a digital intervention before the minimum number of modules that are expected to lead to clinical benefits have been completed—may also impede mental health benefits that could potentially be gained from DMHIs [11]. For these reasons, solutions for optimizing user engagement with DMHIs represent a critical step toward advancing the wider integration of digital interventions in routine practices of health, education, and community settings where they can reach and benefit young people.

To date, there is inconclusive evidence supporting the efficacy of technology-supported strategies (eg, reminders, coaching, and personalized feedback) toward improving engagement with DMHIs [12]. It has been hypothesized that the lack of efficacy associated with these engagement strategies may be due to poor alignment with the needs and preferences of users [13]. Indeed, there is little evidence that such engagement strategies have been co-designed with, or incorporated the perspectives of, those who are intended to benefit from DMHIs [4]. To improve the effectiveness of both DMHIs and any supporting engagement strategies, adopting user-centered design approaches—such as participatory design or co-design—has been strongly recommended [14]. While several studies have now described processes of actively involving service users in the development of mental health–focused digital interventions for youth and young people [4,15], no studies have yet discussed such processes in the context of adjunctive strategies—strategies that are accessed externally to DMHIs (eg, via social media or the internet) but are designed to be used in conjunction with the latter—for promoting engagement with digital interventions.

This study details the development of a technology-supported adjunctive strategy for increasing engagement with LifeBuoy [16]—a smartphone app designed to help young people manage suicidal thoughts. LifeBuoy seeks to achieve this by imparting distress tolerance and emotional regulation skills that are grounded in dialectical behavior therapy and acceptance and commitment therapy.

A recent evaluation of LifeBuoy supported the app’s efficacy in reducing the severity of suicidal ideation but not other outcomes of interest such as depression, anxiety, or general psychological distress [16]. Additionally, while module completion rates were high, most participants stopped engaging with the app after a month [16]. By adding an adjunctive strategy to reinforce therapeutic content and enhance sustained engagement with the app, we hoped to see improvements across both mental health and suicidality-related outcomes.

Our design approach integrated existing literature with qualitative insights obtained from young people with lived experience of suicide. This study is the first to discuss how user-focused design practices may be applied in developing strategies for promoting engagement with DMHIs for young people.

## Methods

### Overview

**Figure 1:**
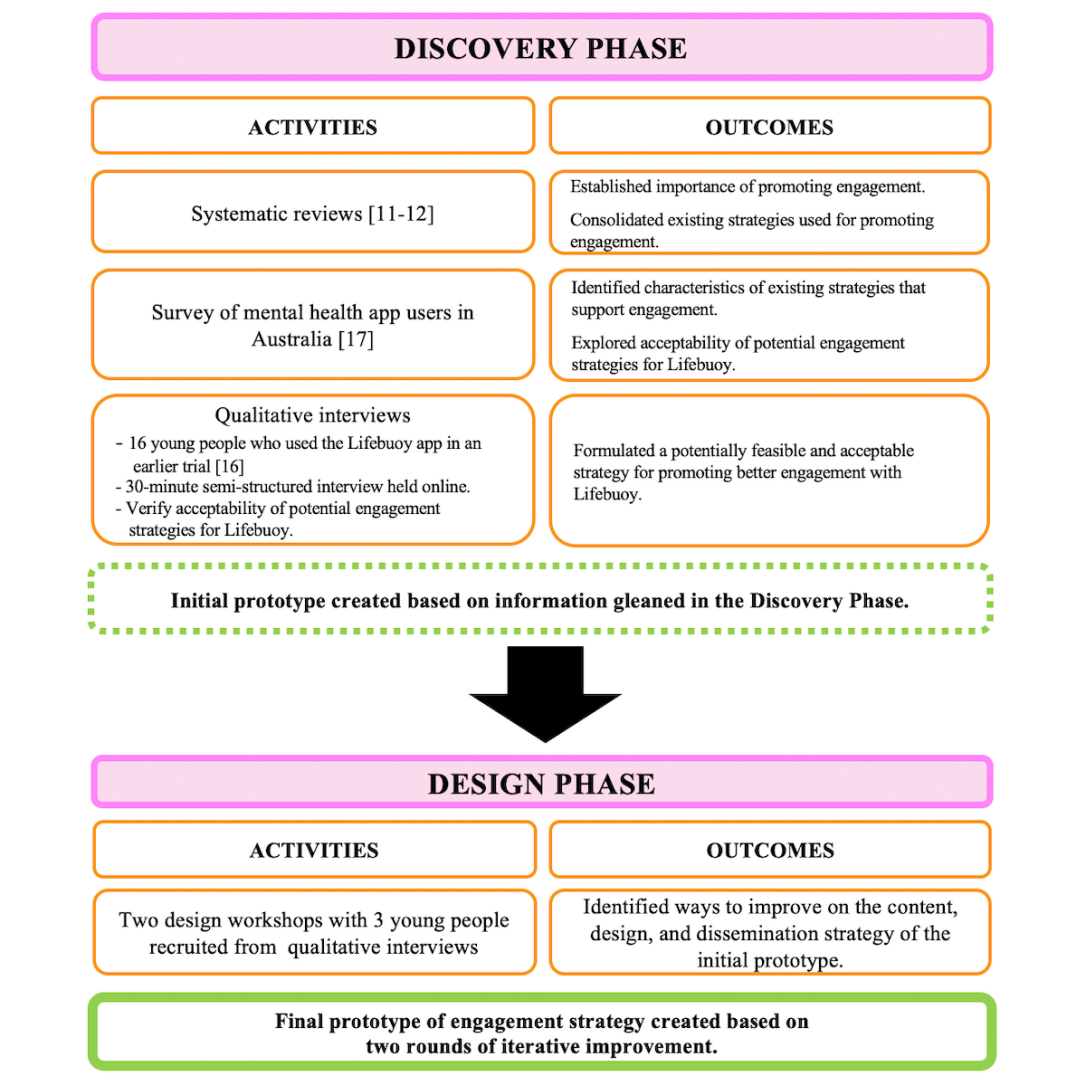
Overview of the approach used in developing the adjunctive strategy for LifeBuoy.

The development process, summarized in Figure 1, consisted of 2 main phases. The aim of the discovery phase was to develop an initial prototype of the engagement strategy. This was carried out by integrating existing evidence from the literature with the perspectives of (1) the broader population of mental health app users and (2) LifeBuoy users. Subsequently, the design phase sought to iteratively refine this initial prototype. This was achieved by collaborating with a smaller group of LifeBuoy users across 2 design workshops, culminating in the final version of the strategy. The sections that follow will focus on the interviews conducted during the discovery phase and the workshops conducted during the design phase.

### Discovery Phase: Development of the Initial Prototype

#### Activities

Three broad activities were conducted in the discovery phase. First, 2 systematic reviews were conducted to (1) clarify the association between engagement and mental health outcomes in the context of digital interventions [11] and (2) explore the range of strategies that have been used to promote engagement with these interventions [12]. The main findings of these reviews have been discussed earlier. Collectively, they highlighted the importance of adopting a user-centered approach in developing strategies for promoting engagement with DMHIs.

Second, a cross-sectional survey of mental health app users in Australia was conducted to gather insights on the feasibility and acceptability of potential strategies for promoting engagement [17]. Key insights that were directly relevant to the development of the engagement strategy for LifeBuoy were that (1) content should be brief, practical, and digestible; (2) the strategy should be visually appealing; and (3) social media might be an acceptable platform for the strategy among young adult users [17].

This paper is focused on describing the third group of activities—semistructured qualitative interviews of LifeBuoy users. The interviews were conducted to assess the acceptability of having an adjunctive strategy and gain users’ perspectives on the specific characteristics (eg, content, design, medium or platform, and frequency of delivery) that this strategy should possess.

#### Interview Participants

A total of 16 young people who had been allocated to the intervention condition in the LifeBuoy trial were invited to take part in the interviews. All participants were recruited on a first-in-first-served basis and met the trial inclusion criteria: (1) being aged 18 to 25 years, (2) having previously experienced suicidal thoughts, (3) being conversant in English, (4) living in Australia, and (5) being a smartphone user.

#### Interview Materials

Interviews were conducted using a semistructured guide developed by LM and MT (see Multimedia Appendix 1). Young people were asked about their views regarding the suitability of possible adjunctive strategies for promoting engagement with LifeBuoy. Based on the existing literature, strategies that have been used to increase engagement with digital interventions for mental health include electronic prompts [18], fun facts [19], and quizzes [20]. These strategies were of interest in this study, as they did not require ongoing human support, consistent with the self-guided delivery model of the LifeBuoy app. In addition, given the widespread use of social media among young Australians [21], interviewees were also asked whether they thought an accompanying social media account would help improve engagement with LifeBuoy.

#### Interview Procedure

After completing the 6-week postintervention survey in the LifeBuoy trial, invitations to participate in a qualitative interview were sent to young people allocated to the intervention condition. Invitation distribution was automated using the Black Dog Institute’s bespoke trial management software.

Young people who expressed interest in being interviewed were subsequently contacted, via email, by a member of the research team to schedule a date and time. They were also provided with a soft copy of the participant information sheet and consent form that was specific to the qualitative interview. Before starting the interview, participants were given a chance to ask any questions about the study and asked to read the declaration of consent aloud to demonstrate opt-in consent.

All interviews were conducted and recorded using Zoom videoconferencing software. Each interview lasted no longer than 30 minutes. Audio recordings of the interviews were transcribed by a professional transcription service.

### Design Phase: Refining the Initial Prototype

#### Activities

Findings from the interviews were analyzed (see Data Analysis section) and integrated with insights from the survey and systematic reviews to create an initial prototype of the engagement strategy. Two design workshops were then conducted to iteratively refine this initial prototype. An agenda for each workshop can be found in Multimedia Appendix 2. Best practices in participatory and co-design methodology—including adopting a design-led approach, sharing decision-making power fairly between researchers and end users, and treating professional expertise and lived experience with equal value [22,23]—were applied to the way in which the workshops were conducted.

#### Workshop Participants

Three young people who took part in the qualitative interviews were invited to participate in the workshops as lived experience advisors. They were invited to these workshops by LM and DZQG based on the depth of insights and expressive communication displayed during the qualitative interviews. Verbal consent to participate was recorded at the first workshop.

#### Workshop Procedure

Before starting the first workshop, advisors were given a chance to ask any questions about the study and read the declaration of consent aloud. To preserve anonymity, advisors were asked to turn off their cameras and change their display names to pseudonyms before audio recording commenced. Both workshops were conducted remotely over Zoom.

The first workshop was focused on the content and aesthetics of the engagement strategy. Discussions were centered on improving the quality and alignment of existing content with the profile of LifeBuoy users. Similarly, advisors were asked for their feedback on the aesthetics of the current prototype. Insights obtained from this workshop were used to revise the initial prototype.

Two weeks before the second workshop, the revised prototype was disseminated to advisors via email. Besides further discussions on the content and design of the revised strategy, the second workshop also focused on the implementation of this strategy. Specific prompts and questions used to elicit discussion can be found in Multimedia Appendix 2.

#### Data Analysis

Thematic analysis was used to analyze qualitative data from the interviews and workshops because of its rigorous methodology and suitability for use in applied research [24,25]. The first step involved familiarization with the data sets and concurrent verification of transcription accuracy by the first author (DZQG). Grammatical and spelling errors for each transcript were corrected as the audio was cross-checked against the transcription. Initial impressions of the data set were then used to generate a list of preliminary codes. Thereafter, an initial coding framework was drafted, and potential themes were generated. An inductive approach was used to identify and organize codes into themes to minimize the potential influence of preconceived opinions.

A second researcher (LM) helped to refine and finalize the coding frame. Consensus on the suggested themes and codes was reached through a discussion between DZQG and LM. In total, 50% of the interview and workshop data sets were double coded by DZQG and LM using the final coding framework, achieving an average agreement of 82%. The remaining transcripts were coded by DZQG. All authors conducted a final evaluation of the alignment between the suggested themes and the codes. NVivo (version 12; QSR International) was used to perform all qualitative analyses.

### Risk Management

Young people who took part in the interviews and workshops were encouraged to inform the researchers verbally, or by private chat message in the videoconferencing platform, if they were feeling distressed, needed a break, wanted the session to be stopped, or wanted to withdraw their participation. In addition, they were informed that they had immediate access to speak with the team clinical psychologist and would be contacted by the team clinical psychologist within 48 hours if they required support. At the end of every interview and workshop, participants were asked if they were feeling distressed and reminded of this support as well as external supports such as their primary health care professional or Lifeline Australia (24-hour crisis contact).

### Ethics Approval

These research activities were conducted in accordance with the Declaration of Helsinki and approved by the University of New South Wales Human Research Ethics Committee (HC190764 and HC200616). Participants for both the semistructured interviews and design workshops were provided with a soft copy of the participant information sheet and consent form at least 2 days before the scheduled research activity. All study data were anonymous. Pseudonyms were used during the interviews and design workshops to ensure protection of the identities of participants. Interview participants each received an AUD $30 (US $20) e-gift card to reimburse them for their time. Lived experience advisors were reimbursed at a rate of AUD $60 (US $40) per hour for time spent during the design workshops and reviewing content shared via email outside of the workshops. This rate was based on the Black Dog Institute’s paid participation policy for individuals with lived experience [26].

## Results

### Characteristics of Interview and Workshop Participants

The 16 participants interviewed were mostly female (n=12, 75%; n=2, 13% male; n=2, 13% nonbinary) and had a mean age of 21.1 (SD 2.49) years. Just over half of the participants (n=9, 56%) identified as belonging to a sexual minority group. All 16 participants reported previously being diagnosed with a mental illness, and 15 (94%) reported receiving prior mental health treatment. In total, 8 participants (50%) reported a history of attempted suicide. Participants completed an average of 5.94 (SD 1.88) modules, with 10 participants (63%) completing all 7 modules. Overall, these demographic, clinical, and intervention usage characteristics were representative of the subsample of participants randomized to the intervention condition (n=228) in the LifeBuoy evaluation study [16]. Workshop participants were 2 women and 1 man (age range 19-24 years). In this study, we did not have any participants reporting distress or requiring follow-up intervention.

### Developing the Initial Prototype

Findings from the interviews and their implications for the design of the engagement strategy are summarized in Table 1.

**Table 1:** Themes and design implications from the qualitative interviews.

Key themes and subthemes		Quotes	Design implications
**1. Characteristics of the adjunctive strategy**			
	Unique or interesting features	*I love learning things … a quiz would be a lot of fun and little fun facts would, stick out more against all the notifications on the phone.* [Participant #1]	Type of content provided, and the way it is presented, needs to capture the interest of users.
	Useful and applicable content	*If it's stuff where it's like, oh, I can actually use that information, or do something with that.* [Participant #5]	Content needs to be well-aligned with users’ needs.
**2. Timing of notifications**			
	Regular, but not too frequent	*I guess personally, I find when I have a reminder on my phone that goes off every day, I quickly start to ignore it. So maybe if it's too consistent, it sort of gets overlooked.* [Participant #15]	Weekly notifications may be most appropriate.
	Type of day	…*maybe weekends, where I don't have other things on.* [Participant #3]	Users may be more available to attend to notifications pushed out on weekends than weekdays.
	Time of day	*I guess probably two times, either in the morning before 9AM, like before I wake up… Or the other time would probably be maybe towards the end of the day. So, maybe like 7-8PM.* [Participant #8]	Mornings and early evenings may be the most suitable times of the day for pushing notifications.
**3. Suitability of social media**			
	Familiarity	*I love a good social media page. I think most people our age probably do.* [Participant #5]	Using Instagram as a platform will reduce the inertia toward users having to learn a new platform.
	Sense of community	*I like when there's sort of a community aspect to it. Liking this page and doing that, it just sort of makes you more involved in the whole process. And it also reminds you if you're just scrolling through Facebook and then you see a post, then it will remind you, oh, maybe I'll use the app now or later because sometimes it's easy to forget.* [Participant #3]	Users may feel more motivated to attend to the strategy and app if they are aware that they are not alone in the challenges they face.

#### Theme 1: Characteristics of the Adjunctive Strategy

Participants agreed that the strategy needed to be able to pique users’ attention and interest and that the best way to achieve this would be to use methods different than conventional notifications or reminders on smartphone apps. These might motivate users to attend to the strategy and, subsequently, engage with the app.

Participants also expressed being more inclined to attend to the strategy if it provided information that they found useful and applicable to their situation. This might then lead them to engage with the app.

#### Theme 2: Timing of Notifications

When asked about their preferred frequency at which notifications should be disseminated, participants expressed preference for receiving them regularly (eg, weekly) but not too frequently (eg, daily). Most of them explained that being notified too frequently could be frustrating or desensitizing and would lead them to ignore notifications.

Some participants expressed that they were more likely to respond to notifications from the adjunctive strategy on weekends, as they were less likely to be busy with other activities compared to weekdays. Other participants felt that they would attend to notifications sent on weekdays if these were sent at the beginning or at the end of their day. Specifically, the time intervals between (1) waking up and before school or working hours and (2) after school or working hours but before bed were the most feasible.

#### Theme 3: Suitability of Social Media

There was strong support for leveraging social media to engage young people with the intervention. All but 2 participants had positive views about using social media as a platform through which the engagement strategy is accessed. This was due to general familiarity with these platforms, as well as prior use of these platforms for the specific purpose of seeking help for mental health needs. Most participants reported that Instagram was their preferred platform.

Furthermore, some participants noted that a social media platform has the potential to foster a sense of community among LifeBuoy users. This would assist with reminding users that they are in the help-seeking process together with others who share their struggles. Engagement with social media will also serve as an indirect reminder for them to check the app out.

### Refining the Initial Prototype

Overall, three main themes emerged over the 2 design workshops, and these themes were centered around (1) characteristics (content and dissemination), (2) aesthetic design, and (3) a component with in-depth information. Subthemes, representative quotes, and design implications are outlined in Table 2.

**Table 2:** Themes and design implications from the design workshops.

Key themes and subthemes		Quotes	Design implications
**1. Content and information provided**			
	Practical and easy to use in everyday life	*I'd probably more likely engage with mindfulness self-care or distress management, maybe breathing as well.* [Advisor #1]	Include simple, time-effective activities that can provide immediate benefits in mental health.
	Uplifting and motivational	*… self-care and self-value, self-worth, belief is something that I just constantly need drilled into me* [Advisor #2]	Include information that encourages and inspires.
		*I really appreciated some of the messages behind the posts…I feel like they would be really uplifting and inspiring, which I think is what we're going for here, which is great...definitely somewhere between corny and inspirational.* [Advisor #3]	Avoid clichéd, idealistic phrases.
	Emotionally validating	*It doesn't even have to necessarily talk about how it (suicidal thoughts) works biologically, but just some info regarding... Even just statistics, the reminder that it is normal to experience these things occasionally. Just a little bit about how many people have it, or ways to cope … any sort of message, whichever way it's put, really needs to have that backing of validation.* [Advisor #1]	Information should resonate with the feelings that users may be experiencing. Avoid presenting facts, tips, or advice in a stoic manner. Instead, wording of content needs to be positive, optimistic, and nurturing.
	Refrain from advertising or hard-selling LifeBuoy	*I think it is very important that we don't come off as a “Here's a corporation that are pretending to care about you” kind of thing, which I know obviously LifeBuoy is not, but it is important to try and not be seen as that.* [Advisor #3]	Avoid language that may come across as promoting the app too strongly.
	Frequency and timing	*I think it's good to have it once a week to remind people that it is important to look after your mental health, but it's not too much that it's like, “My gosh, I'm being bombarded with having to look after myself”.* [Advisor #3] *I think 4:00(pm) till 7:00(pm) is good. I do believe that after a certain time, you've already sort of wound down. You're not going to get engagement. I certainly don't want to engage after a certain time of the night.* [Advisor #1]	Weekly notifications may have a greater likelihood of being attended to if sent in the late afternoon or early evening on weekdays, once a week.
**2. Aesthetic design**			
	Calming	*I feel like nature's always one of those things that a lot of people find it kind of rejuvenating to see … a simple aesthetic stock photo would be my favourite.* [Advisor #3] *The nature ones would be good for breathing and mindfulness… the colors were nice. I thought the light blue was really calming to come into.* [Advisor #1]	Use backgrounds that users find calming; for instance, nature-themed backgrounds with less vibrant colors or a water-themed color palette.
	Clear to read	*Instead of just having the plain background, there's like a tiny, semi-transparent white text box behind the actual text, which I think makes a text a lot easier to read. It doesn't overload your brain on trying to find where the words are. [Advisor #3]*	Ensure sufficient color contrast between words and their background for easier reading.
**3. Option for more detailed content**			
	Preference for more information	*I agree, it (blog) would be a great resource to be able to go to if you wanted to know more, if you finished ‘Isle Know-It-All’ (one of the islands in the LifeBuoy app) in two seconds and you're like, “Oh, I don't know at all yet. I want to know more”.* [Advisor #2]	Cater to users with greater information needs.
	Targeted advice on help-seeking	*One topic that really stands out for me, that goes in line with the posts that are already there, would be what would happen if you were to tell your psychologist that you have suicidal thoughts or I have suicidal ideation and it's scaring me, sort of thing, what do I do about it?* [Advisor #1]	Provide tips for first-time therapy seekers on what to expect and how to manage their suicidal feelings.

#### Theme 1: Characteristics of the Engagement Strategy

Advisors expressed a preference for content that was simple and actionable. They shared that, during times of acute psychological distress, they found it very difficult to consciously and independently engage with complex methods of coping. Simple strategies with clear instructions might therefore be the best way of helping young people tide over more intense periods of distress.

Motivational and uplifting messages would be helpful for reminding users that their well-being is important and encouraging them in their everyday life. Advisors felt that these messages should be focused on encouraging young people to persevere in their life challenges. In doing so, the messages should not come across as clichéd, patronizing, or overly idealistic.

Content should also be emotionally validating. Advisors emphasized that the general tone of any wording should be gentle and emotionally supportive. In addition, they recommended that some content be designed specifically to validate negative emotions that young people might be experiencing, as they may not have had opportunities to express these feelings. They suggested that one way this could be done was to provide information, such as prevalence statistics, that reminds users that they are not alone in their struggles.

Advisors stressed the importance of ensuring that content not be perceived by users as advertising or hard-selling the LifeBuoy app. They felt that users who developed such perceptions would significantly be less willing to engage with both the strategy and the app. According to advisors, this concern stems from a broader discontentment among their contemporaries with the way corporations have used social media to promote their products. Thus, it was imperative that messages convey genuine concern for the well-being of users.

Regarding the frequency and timing of dissemination of content, weekly updates were considered sufficient to keep users reminded of the LifeBuoy app. Advisors expressed that they were more likely to attend to Instagram notifications sent in the late afternoon to early evening. This time window was perceived to be suitable as it represents a block of time after the end of school and work but before bedtime.

#### Theme 2: Aesthetics and Design

Advisors unanimously supported the use of nature-themed backgrounds. They felt that the nature-themed backgrounds would be better paired with the content topics and provided consistency with the marine-themed design of the LifeBuoy app. Aesthetic features of the posts could also help to reduce feelings of distress by having a calming effect on mood. Advisors also recommended improving the visibility of text-based content by increasing the color contrast between the text and the background. Specifically, they suggested placing text-based content in front of a white background to enhance readability. Finally, advisors expressed that a key reason why Instagram was more suitable than platforms such as Facebook was because Instagram was better at delivering information in a visually appealing way.

#### Theme 3: Component With More Detailed Content

During the workshops, advisors reflected on the possibility that some users, like themselves, may be avid information seekers who appreciate more in-depth content. They also felt that certain pertinent topics should feature in the content of the strategy. In particular, they explained that a topic on help-seeking for suicide ideation would be very relevant for users seeking therapy for the first time. Content on such a topic might not be very helpful for users if it were too concise. Advisors concurred that social media platforms might not be an ideal way of presenting topics that contain more comprehensive information. In the discussions, a spontaneous suggestion about having a blog as one component of the adjunctive strategy was well-received by advisors.

### Initial, Revised, and Final Design Samples of the Engagement Strategy Through the Design Process

**Figure 2:**
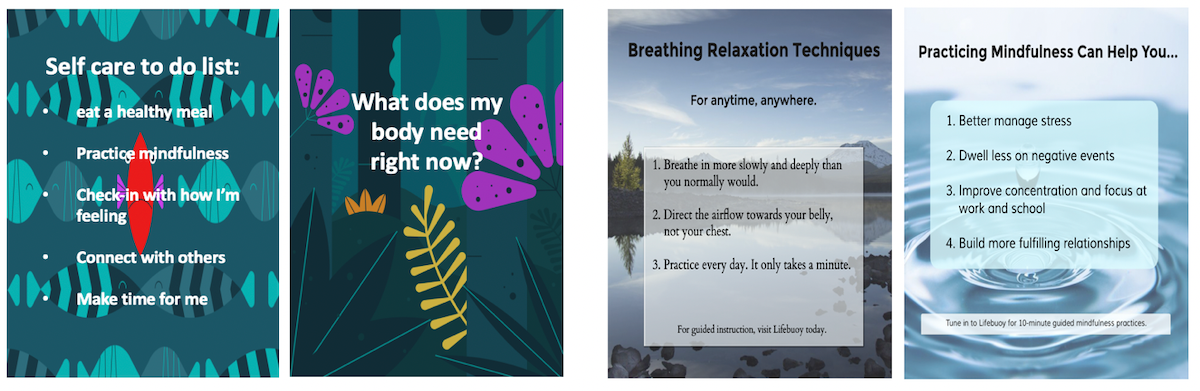
Initial Instagram designs were generated based on the qualitative interviews, using LifeBuoy app–themed (left) versus stock image–themed backgrounds (right).

**Figure 3:**
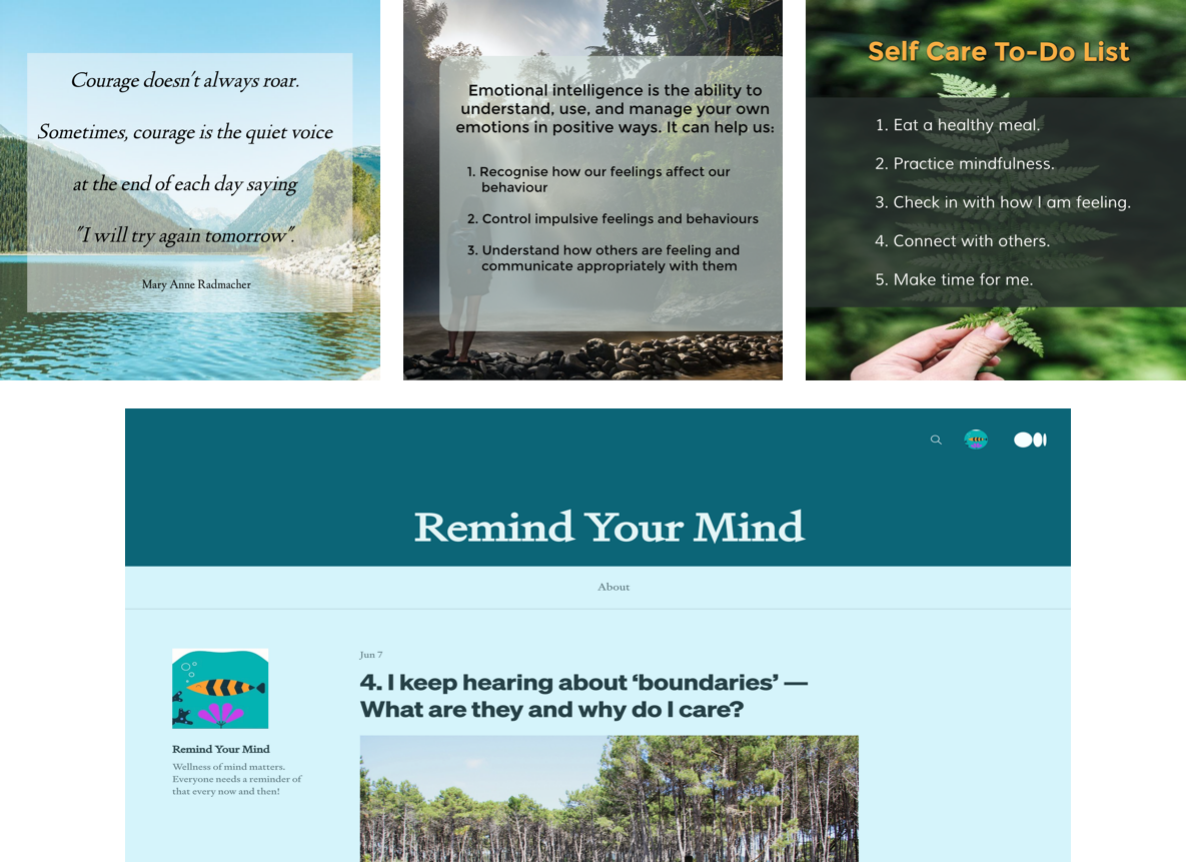
Samples of the revised Instagram posts (above) and initial blog design (below), following feedback from the first workshop.

**Figure 4:**
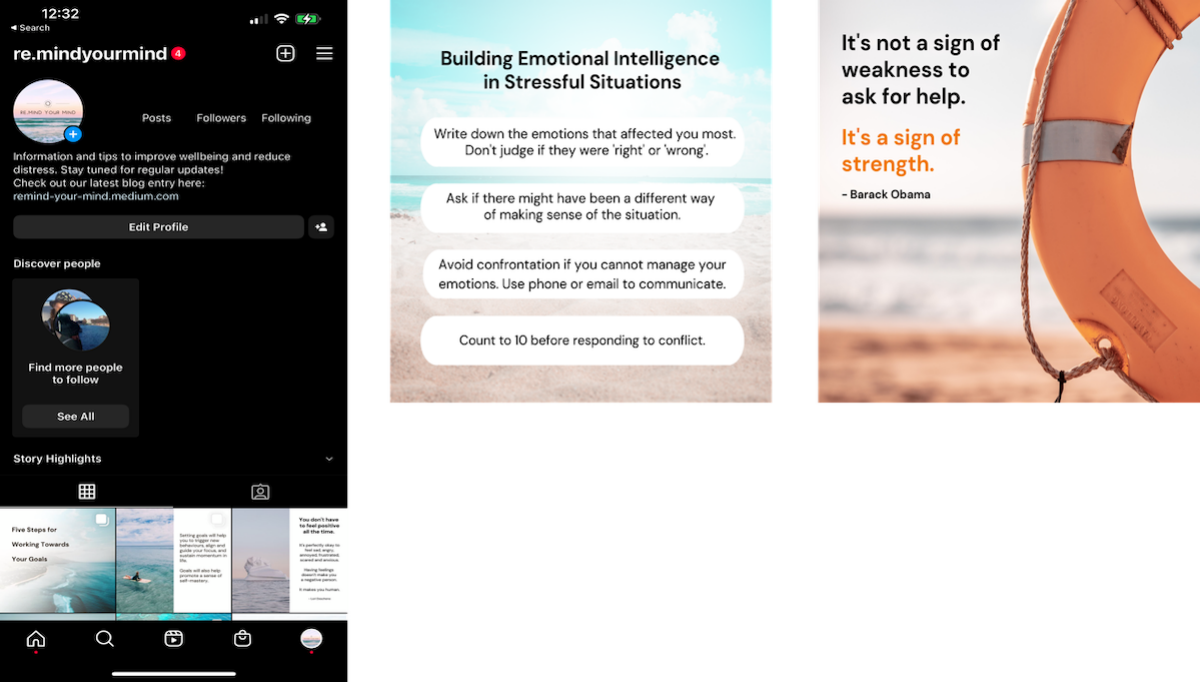
Final Instagram design: landing page (left), sample informational post (center), and sample inspirational post (right).

**Figure 5:**
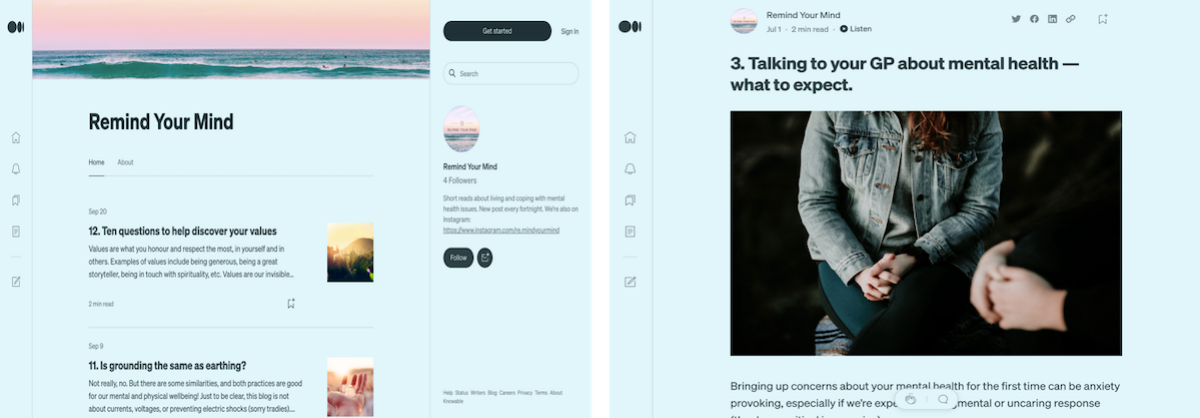
Final blog design: landing page (left) and sample post (right).

Sample social media posts and blog posts at different stages of the design process are summarized in Figures 2 to 5. Based on insights from the qualitative interviews, sample Instagram posts, which were informative, uplifting, and aligned with content in LifeBuoy were created to start discussions at the first workshop (Figure 2). Several variations of each post were created using different types of color schemes, aesthetics (eg, nature and baby animals), and fonts, with the objective of getting feedback from workshop participants about their preferred styles.

Feedback and insights from the workshops were used to refine the initial samples before the second workshop (Figure 3). Motivational posts were worded to read as encouraging but realistic. Psychoeducational posts were written in simple and clear language. Instructional posts were presented in bite-sized pieces of information with succinct steps provided. A blog was also created. A water-themed nature background was used for the blog to align its design with that of the LifeBuoy app and with the preferences of young people. The blog posts provided detailed information on a range of topics related to mental well-being and help-seeking. They were written by a trained clinical psychologist (LM) or young adults with lived experience of suicide. Each post had an estimated reading time of 2 to 4 minutes.

The final version of the engagement strategy was created from the initial prototype following 3 rounds of iterative refinements arising from the workshops. It consisted of three main components: (1) an Instagram account (Figure 4) containing posts that delivered concise informational or inspirational content aligned with the content in the app, (2) a blog (Figure 5) with entries containing more detailed and in-depth therapeutic information and advice for maintaining mental wellness, and (3) weekly emails inviting users to visit these platforms. A total of 42 Instagram posts and 12 blog articles were created, collectively allowing for new material to be posted every 2 to 3 days over the course of the trial. To balance safety considerations and ensure the strategy required only minimal ongoing human support, user comments were disabled for both Instagram and the blog. To ensure that users could access Instagram content anonymously, the account was made public instead of private. The like count was also hidden to preserve the identity of users who liked any of the Instagram posts.

## Discussion

### Principal Findings

This paper described the qualitative processes and activities undertaken with young people who had a lived experience of suicidal ideation to develop an adjunctive strategy aimed at promoting engagement with the LifeBuoy smartphone intervention. Social media, particularly Instagram, was seen as an acceptable medium to deliver brief, practical, and inspirational content that could motivate and encourage young people to engage with the intervention. A blog with articles contributed by a clinician and people with lived experience of suicide was identified as the preferred medium to deliver in-depth information on (1) living with and (2) seeking help for suicidal thoughts. Nature-themed visuals with calming colors were used as the design theme.

### Comparison With Previous Literature

To date, no study has reported on the process of designing adjunctive strategies for supporting engagement with DMHIs. Notwithstanding, our study findings were generally consistent with past research on the co-design of digital interventions for younger users, which found young people to have a preference for direct, practical information [27], content that positively impacts mood (such as relaxation techniques, distraction activities, or uplifting quotes) [23], and designs that are visually appealing [28].

Nature-themed backgrounds appealed most to advisors because of their calming effect on mood. These views are congruent with theories and findings from environmental psychology. According to Stress Reduction Theory [29], exposure to nature reduces stress and has therapeutic effects on both physiological and psychological well-being. Importantly, earlier research has extended the postulations of Stress Reduction Theory by demonstrating that virtual exposure to natural environments can be effective toward reducing stress and attenuating subsequent reactivity toward stressful stimuli [30]. This is aligned with feedback from the design workshops that looking at nature-themed stock photos has a rejuvenating effect.

The young people who were interviewed felt that Instagram was a suitable social media platform for the adjunctive strategy. This was attributed to their existing familiarity with the platform and the perceived sense of community support provided by other users. This finding appears to contradict previous research reporting an inverse association between Instagram use and mental health among youth and young adults [31,32]. However, a recent study found that Instagram use had an indirect effect on reducing suicidal ideation through the mediating effect of perceived burdensomeness among young people aged 18-25 years [33]. Although more research is needed to elucidate the relationship between Instagram use and mental health, these findings collectively suggest that an accompanying Instagram account might be a suitable complement to DMHIs targeting suicide ideation among young people.

Currently, there are no studies promoting engagement with digital interventions, which have examined the feasibility or acceptability of adjunctive digital strategies that provide detailed content on topics relevant to users [12,18]. Using a blog emerged as a novel way to provide in-depth information on topics of interest to young people with suicidal ideation and reinforce the therapeutic content covered in LifeBuoy. Importantly, the blog allowed young people with lived experience of suicide to directly contribute content that would likely resonate with the needs and challenges faced by intended users of the LifeBuoy app. It is also hoped that this would increase the acceptability of the blog among young people who use LifeBuoy. Nevertheless, whether the blog is efficacious toward promoting engagement with LifeBuoy requires empirical investigation.

### Strengths

As far as the authors are aware, this study is the first to discuss the application of a participatory design approach in the context of developing an adjunctive strategy to complement a digital intervention for mental health. The workshop aspect of the design process drew from principles and recommendations of participatory and co-design frameworks [22,34]. Young people’s perspectives on what facilitates engagement with DMHIs were generally consistent with the extant literature.

A rigorous methodological approach integrating evidence, professional input, and lived experience insights was used in the planning, design, and refinement of the adjunctive strategy. The rationale for developing the strategy was grounded in evidence from the research literature and perspectives of the broader mental health app user population in Australia. Young persons who contributed their insights during the interviews and workshops came directly from the target population that the app was designed for. Development of the initial prototype was based on data from the qualitative interviews. Finally, the initial, revised, and final versions of the adjunctive strategy were developed in an iterative manner between each round of design activities. Although only 2 workshops were conducted, this was sufficient given that a high degree of consensus between advisors and researchers regarding the strategy had been reached by the second workshop.

A robust coding frame was developed to consolidate and make sense of the feedback obtained. In total, 50% of all qualitative data were double coded, and a reasonable overall level of intercoder agreement was achieved even though this was not an essential requirement for thematic analysis [35].

Finally, this work is well-aligned with high-level national [36] and international [37] efforts aimed at supporting suicide prevention by empowering young people with lived experience to play a greater role in the creation and dissemination of mental health services.

### Limitations

This study had several limitations. Only the research team was involved in every phase of this project. As such, they may invariably have shaped some aspects of the strategy’s development. For example, as described in the Methods section, decisions on the types of engagement strategies to explore further during the interviews and workshops were made before users were involved. Nonetheless, it was necessary and pragmatic to streamline the qualitative data collection to focus on solutions that were feasible based on existing resources. However, these decisions were informed by the existing literature and are therefore consistent with what young people have identified as potentially useful engagement strategies. Moreover, all suggestions provided by young people were fully considered. In other words, the eventual version of strategy was based not only on their perspectives of what might work but also on what might not work.

Resource constraints affected the implementation of development activities in several ways. Workforce and financial constraints did not allow for multiple parallel workshops to be conducted with different groups of young people. Doing so may have generated additional insights that could inform the development of the adjunctive strategy. Finally, operational constraints associated with technical expertise and funding meant that not all feedback from the design activities could be incorporated into the adjunctive strategy. However, the research team was transparent with participants by informing them that not all the feedback provided could feasibly be acted on.

Characteristics relating to the study sample may have restricted the scope of design activities conducted, and consequently, the insights obtained. Young people who took part in the interviews represented only a small proportion of all LifeBuoy users (n=228) [16]. Notwithstanding, the demographic and clinical profiles of young people involved in this study were similar to the wider sample. Finally, only potential end users of LifeBuoy were involved in the design process. Engaging other stakeholders, such as mental health professionals or caregivers, may have yielded useful perspectives that could have contributed positively to the development of the strategy.

### Conclusions and Future Directions

This study is the first to thoroughly describe the process of designing an adjunctive strategy for promoting engagement with a digital intervention. Characteristics pertaining to the platform, content, aesthetics, and dissemination of this strategy were based on integrating past literature with insights from young people with lived experience of suicide. The design approach, its associated activities, and how the findings were used toward creating and refining the adjunctive strategy were documented in detail. The work undertaken in this study may be helpful for informing subsequent projects aimed at creating resources to support engagement with digital interventions for suicide prevention or mental health.

The next step for this research will be to explore the acceptability and satisfaction of the adjunctive strategy in a large sample of young people who fit the intended user profile for LifeBuoy. This step is important for confirming the representativeness of end-user feedback received during the design process and provides an opportunity to identify and improve upon any potential limitations in the strategy before efficacy testing.

Future efforts should strive to embrace a co-design approach in developing adjunctive strategies for promoting engagement with digital interventions. Co-design is one of the most important emerging concepts in modern health care research because of its focus on addressing gaps in the availability of mental health–related services that are appropriate, acceptable, and relevant to priority populations [38]. Empowering users to be equal partners in the design and development processes may be crucial for improving engagement with mental health interventions which, thus far, has been tied to lack of relevancy or mismatch in needs.

## Appendix

Multimedia Appendix 1 Interview guide.

Multimedia Appendix 2 Workshop guide.

## Abbreviations

DMHI: digital mental health intervention

## Data Availability

The data sets generated or analyzed during this study are not publicly available due to the sensitive nature of the data but are available from the corresponding author upon reasonable request.

## References

[1] (2021). Suicide worldwide in 2019: global health estimates. World Health Organization.

[2] (2021). Deaths by suicide among young people. Australian Institute of Health and Welfare.

[3] Cadigan JM, Lee CM, Larimer ME (2019). Young adult mental health: a prospective examination of service utilization, perceived unmet service needs, attitudes, and barriers to service use. Prev Sci.

[4] Bevan Jones R, Stallard P, Agha SS, Rice S, Werner-Seidler A, Stasiak K, Kahn J, Simpson SA, Alvarez-Jimenez M, Rice F, Evans R, Merry S (2020). Practitioner review: co-design of digital mental health technologies with children and young people. J Child Psychol Psychiatry.

[5] Lehtimaki S, Martic J, Wahl B, Foster KT, Schwalbe N (2021). Evidence on digital mental health interventions for adolescents and young people: systematic overview. JMIR Ment Health.

[6] Torok M, Han J, Baker S, Werner-Seidler A, Wong I, Larsen ME, Christensen H (2020). Suicide prevention using self-guided digital interventions: a systematic review and meta-analysis of randomised controlled trials. Lancet Digit Health.

[7] Fleming T, Bavin L, Lucassen M, Stasiak K, Hopkins S, Merry S (2018). Beyond the trial: systematic review of real-world uptake and engagement with digital self-help interventions for depression, low mood, or anxiety. J Med Internet Res.

[8] Karyotaki E, Kleiboer A, Smit F, Turner DT, Pastor AM, Andersson G, Berger T, Botella C, Breton JM, Carlbring P, Christensen H, de Graaf E, Griffiths K, Donker T, Farrer L, Huibers MJH, Lenndin J, Mackinnon A, Meyer B, Moritz S, Riper H, Spek V, Vernmark K, Cuijpers P (2015). Predictors of treatment dropout in self-guided web-based interventions for depression: an 'individual patient data' meta-analysis. Psychol Med.

[9] Välimäki M, Anttila K, Anttila M, Lahti M (2017). Web-based interventions supporting adolescents and young people with depressive symptoms: systematic review and meta-analysis. JMIR Mhealth Uhealth.

[10] Garrido S, Millington C, Cheers D, Boydell K, Schubert E, Meade T, Nguyen QV (2019). What works and what doesn't work? A systematic review of digital mental health interventions for depression and anxiety in young people. Front Psychiatry.

[11] Gan DZQ, McGillivray L, Han J, Christensen H, Torok M (2021). Effect of engagement with digital interventions on mental health outcomes: a systematic review and meta-analysis. Front Digit Health.

[12] Gan DZQ, McGillivray L, Larsen ME, Christensen H, Torok M (2022). Technology-supported strategies for promoting user engagement with digital mental health interventions: a systematic review. Digit Health.

[13] Mohr DC, Schueller SM, Tomasino KN, Kaiser SM, Alam N, Karr C, Vergara JL, Gray EL, Kwasny MJ, Lattie EG (2019). Comparison of the effects of coaching and receipt of app recommendations on depression, anxiety, and engagement in the IntelliCare platform: factorial randomized controlled trial. J Med Internet Res.

[14] Ioannidis JPA (2016). Why most clinical research is not useful. PLoS Med.

[15] Orlowski SK, Lawn S, Venning A, Winsall M, Jones GM, Wyld K, Damarell RA, Antezana G, Schrader G, Smith D, Collin P, Bidargaddi N (2015). Participatory research as one piece of the puzzle: a systematic review of consumer involvement in design of technology-based youth mental health and well-being interventions. JMIR Hum Factors.

[16] Torok M, Han J, McGillivray L, Wong Q, Werner-Seidler A, O'Dea B, Calear A, Christensen H (2022). The effect of a therapeutic smartphone application on suicidal ideation in young adults: findings from a randomized controlled trial in Australia. PLoS Med.

[17] Gan DZQ, McGillivray L, Larsen ME, Torok M (2023). Promoting engagement with self-guided digital therapeutics for mental health: insights from a cross-sectional survey of end-users. J Clin Psychol.

[18] Alkhaldi G, Hamilton FL, Lau R, Webster R, Michie S, Murray E (2016). The effectiveness of prompts to promote engagement with digital interventions: a systematic review. J Med Internet Res.

[19] Yardley L, Morrison L, Bradbury K, Muller I (2015). The person-based approach to intervention development: application to digital health-related behavior change interventions. J Med Internet Res.

[20] Brown M, O'Neill N, van Woerden H, Eslambolchilar P, Jones M, John A (2016). Gamification and adherence to web-based mental health interventions: a systematic review. JMIR Ment Health.

[21] (2021). Communications and media in Australia: the digital lives of younger Australians. Australian Communications and Media Authority.

[22] Hagen P, Collin P, Metcalf A, Nicholas M, Rahilly K, Swainston N (2012). Participatory Design of Evidence-Based Online Youth Mental Health Promotion, Intervention and Treatment.

[23] Thabrew H, Fleming T, Hetrick S, Merry S (2018). Co-design of eHealth interventions with children and young people. Front Psychiatry.

[24] Braun V, Clarke V (2006). Using thematic analysis in psychology. Qual Res Psychol.

[25] Braun V, Clarke V (2014). What can "thematic analysis" offer health and wellbeing researchers?. Int J Qual Stud Health Well-being.

[26] (2020). Lived experience paid participation policy. Black Dog Institute.

[27] Bevan Jones R, Thapar A, Rice F, Beeching H, Cichosz R, Mars B, Smith DJ, Merry S, Stallard P, Jones I, Thapar AK, Simpson SA (2018). A web-based psychoeducational intervention for adolescent depression: design and development of MoodHwb. JMIR Ment Health.

[28] Stoyanov SR, Zelenko O, Staneva A, Kavanagh DJ, Smith C, Sade G, Cheers J, Hides L (2021). Development of the Niggle app for supporting young people on their dynamic journey to well-being: co-design and qualitative research study. JMIR Mhealth Uhealth.

[29] Ulrich RS, Simons RF, Losito BD, Fiorito E, Miles MA, Zelson M (1991). Stress recovery during exposure to natural and urban environments. J. Environ. Psychol.

[30] Parsons R, Tassinary LG, Ulrich RS, Hebl MR, Grossman-Alexander M (1998). The view from the road: implications for stress recovery and immunization. J. Environ. Psychol.

[31] Faelens L, Hoorelbeke K, Soenens B, Van Gaeveren K, De Marez L, De Raedt R, Koster EH (2021). Social media use and well-being: a prospective experience-sampling study. Comput Hum Behav.

[32] Frison E, Eggermont S (2017). Browsing, posting, and liking on Instagram: the reciprocal relationships between different types of instagram use and adolescents' depressed mood. Cyberpsychol Behav Soc Netw.

[33] Unruh-Dawes EL, Smith LM, Krug Marks CP, Wells TT (2022). Differing relationships between Instagram and Twitter on suicidal thinking: the importance of interpersonal factors. Soc Media Soc.

[34] Dimopoulos-Bick TL, O'Connor C, Montgomery J, Szanto T, Fisher M, Sutherland V, Baines H, Orcher P, Stubbs J, Maher L, Verma R, Palmer VJ (2019). “Anyone can co-design?”: a case study synthesis of six experience-based co-design (EBCD) projects for healthcare systems improvement in New South Wales, Australia. Patient Exp J.

[35] Braun V, Clarke V (2019). Reflecting on reflexive thematic analysis. Qual Res Sport Exerc Health.

[36] (2021). National mental health and suicide prevention plan. Australian Government.

[37] (2022). Preventing suicide: LIVE LIFE implementation. World Health Organization.

[38] Mulvale A, Miatello A, Hackett C, Mulvale G (2016). Applying experience-based co-design with vulnerable populations: lessons from a systematic review of methods to involve patients, families and service providers in child and youth mental health service improvement. Patient Exp J.

