# Supplementary material for: Promoting Engagement With Smartphone Apps for Suicidal Ideation in Young People: Development of an Adjunctive Strategy Using a Lived Experience Participatory Design Approach
Source: JMIR Form Res. 2023 Jun 6;7:e45234. doi: 10.2196/45234 (PMC10282914; doi:10.2196/45234)
Supplement: Multimedia Appendix 1 [file formative_v7i1e45234_app1.docx]

**Multimedia Appendix 1: Interview guide**

**Introduction**

“Good morning/afternoon/evening. Thanks for taking the time to join our interview about your experience using the LifeBuoy app. My name is ___. I will serve as the moderator for today’s interview. We also have ___, who is a clinical psychologist on site/available by phone during the entire session in case any of you feel distressed. The purpose of today’s discussion is to know more about your user experience with and opinion on the LifeBuoy app.

There are no right or wrong answers to the questions I am about to ask. Please feel free to share your point of view. Everything you say here is confidential. I will be taking notes to help us remember what is said. I am also tape recording the session because we don’t want to miss any of your comments. Please note that audio recordings will be provided to an external agency for transcription. However, no individually identifying information (i.e., names, contact numbers) will be provided to them, so you can not be identified.

Please read the participant information sheet carefully and let me know if you have any questions. [Pause] Do you have any questions? [If participant expresses wish to continue] I will start recording our conservation. Is this fine with you? Please provide your oral consent by reading aloud the statements on the second page of the participant information sheet.”

**Interview Questions**

1. “Please tell me how you feel about the app in general.”
2. “Please tell me the top three most appealing parts of the app to you. Why?” “Is there anything that you don’t like? Why?”
3. “In which scenario, do you find the app useful?” “What would stop you from using the app?” “Do you find the reminder messages helpful?”
4. How do you think we can make the reminders more effective to encourage continued use of the app? For example:

- Are there specific days, or times during a day, when you would be more likely to read the email reminder and use the app after?
- Instead of a reminder, do you think other types of content would have motivated you to use the app (such as a fun fact, quiz, or information about your recent progress)?

1. “How did you feel about the design (colour, illustration)?”
2. “What features would you like to be included in/ excluded from the app?”
3. “What are some of the annoying things you experience in using the app (e.g. internet access, registration)?”
4. Suppose we had a social media (e.g. Instagram; Facebook) page for Lifebuoy with new content uploaded weekly. Would you be keen to follow it, and do you think it would encourage you to use the app? Why and/or why not?

**Exit questions**

“Do you have any comments about the app that you didn’t get a chance to speak about?”

Was there anything about today’s interview that has left you feeling at all upset about, or out of sorts? If so, please let me know and the team clinical psychologist will be available to contact you.

If you’d rather not speak to one of the research team, contact details for independent support organisations are provided on the participant information sheet.”
