# Supplementary material for: Promoting Engagement With Smartphone Apps for Suicidal Ideation in Young People: Development of an Adjunctive Strategy Using a Lived Experience Participatory Design Approach
Source: JMIR Form Res. 2023 Jun 6;7:e45234. doi: 10.2196/45234 (PMC10282914; doi:10.2196/45234)
Supplement: Multimedia Appendix 2 [file formative_v7i1e45234_app2.docx]

**Multimedia Appendix 2: Workshop Guide**

Workshop 1

| **Broad activity** | **Details** | **Time allocated** |
| --- | --- | --- |
| Welcome | 1. Welcome participants and thank them for their time.  2. Remind that session will be recorded. Ask them to change their display names.  3. Confirm that all have read the PISCF. Ask if any final questions about study participation.  4. Seek permission to start recording. | 5min |
| Workshop Aims | Briefly share:  1. Objective of the workshops.  2. What co-design is and what it means for us in adopting this methodology for the workshops.  3. How these workshops fit into the bigger LifeBuoy project. In so doing, state where the project currently is, and where it is headed. | 5min |
| Workshop Ground Rules and House Keeping | 1. Ground rules for workshops  - no right or wrong responses  - please be honest  - feel free to disagree, but be respectful  - feel free to tell the facilitator if your views have been overlooked or misinterpreted  - confidentiality: don’t share what is discussed without permission.  2. Highlight that not all suggestions may be eventually adopted due to budget or technological constraints  3. Remind them that help is available if they feel distressed at any point during, or after the workshops.  4. Feel free to excuse themselves from discussions for a moment if needed (e.g., bathroom break, make a cup of tea).  5. Ask if there are questions before moving to the discussions. | 5min |
| Discussion 1:  Message Content | Provide context to the pre-workshop content—based on feedback from:   - other LifeBuoy users, and - online survey of >200 mental health app users.   Discussion of the two questions on Message Content. Max. 25 mins | 25min |
| Break | Ask participants if they’d like a 5 min break | 5min |
| Discussion 2:  Message Design | Discussion on message designs. Max. 25 mins  Ask about suitability of different types of backgrounds for emails and/or Instagram posts | 25min |
| Wrap up | Summarise feedback for each discussion.  Ask if there was anything that participants felt might be important, but was not discussed? If applicable, put on the agenda for next workshop. | 10min |

Workshop 2

| **Broad activity** | **Details** | **Time allocated** |
| --- | --- | --- |
| Welcome | 1. Welcome participants and thank them for their time.  2. Remind that session will be recorded. Ask them to change their display names to the pseudonym they used in the first workshop.  3. Confirm that all participants are agreeable to take part in today’s workshop.  4. Seek permission to start recording. | 5min |
| Recap | Briefly recap:  1. What co-design is, and what it should looks like as we work together.  2. Ground rules and expectations that were covered in the first workshop. | 5min |
| Discussion 1:  Instagram account | Specific points:  1. Overall suitability.  2. Most and least favourite posts, and reason(s) why.  3. Other types of posts that would be good?  4. Feasibility of Instagram stories.  5. Good examples of Instagram account(s) with a mental health focus that we might learn from? | 25min |
| Discussion 2:  Blog | Specific points:  1. Overall suitability.  2. What other topics do you think would be helpful?  3. Is the interface easy to use, and the aesthetics sufficiently pleasing?  4. Good examples of blogs with a mental health focus that we might learn from?  5. Ask advisors if they might be keen to contribute an article to the blog. | 25min |
| Discussion 3:  Dissemination of Instagram and blog content | Specific points:  1. Preferred format/medium of notification.  2. Frequency.  3. Day(s) of week and time(s) of day that users might be most likely to read and respond. | 20min |
| Wrap up | DG to summarise feedback for each discussion.  Ask if there was anything that participants felt might be important but was not discussed. | 10min |
